# Toward a human-centered use of technology: a stakeholder analysis of harm reduction and CBO staff

**DOI:** 10.1186/s12954-020-00422-y

**Published:** 2020-10-19

**Authors:** Ian David Aronson, Alex S. Bennett, Robert Freeman

**Affiliations:** 1 Digital Health Empowerment, Brooklyn, USA; 2 New York University, School of Global Public Health, New York, USA

**Keywords:** Technology, Harm reduction, Stakeholder, PWUD, COVID-19

## Abstract

**Background:**

Technology can enable syringe service programs (SSPs) and other community-based organizations (CBOs) operating under a harm reduction framework to work with an increased number of clients and can also enable organizations to offer services more effectively (e.g., offering HIV testing in ways participants may be more likely to accept). In the current time of COVID-19 social distancing, technology can also help organizations more safely provide services to people with compromised immune systems and to clients who might otherwise not be reached. However, technology projects implemented in harm reduction settings are frequently conceptualized and developed by researchers or technology specialists rather than by SSP staff or clients.

**Methods:**

To more effectively meet the needs of SSPs and other CBOs across the USA, our team conducted qualitative interviews with 16 individuals who have extensive backgrounds working in the field of harm reduction. Interviews were digitally recorded and professionally transcribed, and the transcripts were checked for accuracy by the interviewers. The resulting transcripts were coded and analyzed to determine emerging themes.

**Results:**

Interviewees mentioned the ability of technology to deliver consistent quality messaging to multiple clients at the same time and the potential to customize or tailor technology-based messaging to specific client populations as positive benefits. Clear barriers to technology use also emerged, in particular regarding privacy, data security, and the need to maintain client trust when discussing sensitive issues (e.g., illicit drug use).

**Conclusions:**

Technology offers the potential to deliver consistently high-quality health communication and maintain contact with clients who may have no other access to care. If designed and managed effectively, technology can also address issues related to providing services during times when physical contact is limited due to COVID-19 social distancing measures.

## Background

After 20 years of tragic, record-breaking overdose deaths [1], widespread HCV infection [2], and the expanding dangers of the novel coronavirus SARS-CoV-2 [3], there is an unparalleled need for community-based care organizations serving vulnerable populations including people who use drugs (PWUD) [4]. At the same time, funding for community-based organizations (CBOs) that provide care to substance users and other underserved populations remains limited, while demand for services can frequently exceed available resources [4]. This may prove especially problematic for organizations serving people who have limited access to health care or who fear stigma and legal issues if they disclose illicit drug use to care providers.

Digital technology, and in particular *custom-authored* digital technology that is designed to meet the needs of specific populations in specified settings, can potentially help CBO staff work with an increased number of clients and provide health communication messaging people can readily understand. In recent years, digital communication technology has been widely used to address health issues among substance using populations. A brief survey of projects includes technology for people who use legal substances (e.g., tobacco [5] and alcohol [6]), illegal substances (i.e., cocaine [7]), and legal substances that are often used in ways other than allowed by law (e.g., prescription opioids that are misdirected or used in greater quantities than prescribed [8]). Digital technology has also been used to address health issues of particular concern to substance using populations (e.g., overdose prevention [9] or HIV/HCV prevention and testing [10]). However, the development of these technologies and accompanying research has been implemented largely in clinical settings. Studies conducted in community settings, even when they involve established CBOs that provide care to PWUD and other underserved populations, are often initiated and led by professional researchers, not CBO staff.

To develop a deeper understanding of ways, digital communication technology can potentially help harm reduction and other community-based organizations deliver services; we conducted a series of interviews detailed in the current paper. In the era of COVID-19, it appears that technology may prove particularly useful in harm reduction settings. (This article uses the Harm Reduction Journal’s definition of “harm reduction”: policies and programs which aim to reduce the health, social, and economic costs of legal and illegal psychoactive drug use without necessarily reducing drug consumption [11]. Likewise, we define “harm reduction settings” as locations in which services are provided to people who use drugs in accordance with the above definition of harm reduction.) While the long-term impacts of SARS-CoV-2 remain unknown, it is clear that social interactions, especially face to face, will be radically transformed as will the nature of service delivery [3, 4]. Thus, it would appear that since the COVID pandemic’s arrival, technology may hold even greater potential to help agencies reach and serve their client base, while maintaining a client-centered framework.

There are multiple ways to create technology-based communications and interventions, ranging from using existing technology (i.e., text messages or a platform like Facebook) to writing new code and custom authoring a new product, as described earlier. We compare the difference to baking a cake from a mix that you buy in the grocery store to making something from scratch: using a mix is easier and takes less time, but when you bake from scratch you know exactly what ingredients you are using, and can customize the recipe to suit your own specific needs. Accordingly, custom authoring requires a specific skill set, which may be aided by an understanding of instructional design principles [12] and multimedia learning theory [13–15], and often requires additional time for extensive testing and revision. It also enables far greater flexibility because writing new code opens up an infinite number of new possibilities, enabling skilled developers to create exactly what they want. This can prove especially valuable when addressing complicated behavioral health issues in challenging settings (e.g., a high-volume drop-in center or an outdoor street outreach site) and when a project requires maintaining the trust and privacy of PWUD clients.

A foundational idea of both instructional design and multimedia learning is that effective uses of technology entail far more than purchasing a device, or a new software package, and turning it on. Instead, the process of developing effective technology entails first conducting a *needs assessment* to determine how computer-based media can be used to most effectively address a specific set of problems in a given environment. Subsequent steps include working with subject matter experts (e.g., syringe service program staff) to determine appropriate outcomes, followed by iteratively drafting solutions that fit technology around an understanding of how the human mind works and how people process new information.

In multimedia learning, this is referred to as a *learner-centered* approach [13], in which new projects are developed around the needs of the intended audience of learners, instead of a *technology-centered* approach in which projects are developed around the affordances of a new technology (i.e., virtual reality is really exciting, so let’s make something that uses virtual reality). The current paper is focused on what we have termed a *human-centered* approach to the use of technology in CBO settings. We use the phrase “human-centered” because we want to create technology that is not only developed around the learners’ needs and cognitive processes, but also focuses on the dignity of people in frequently stigmatized populations [16, 17] and emphasizes content that underscores the importance of compassion for people who may be discriminated against in healthcare and criminal justice settings [17, 18].

To the best of our knowledge, this is the first study to conduct a needs assessment that is guided by theories of instructional design and multimedia learning, and executed within a harm reduction context. We suggest this methodology can potentially lead to more valuable applications of technology because the design process will start with, and build upon, assessments of what CBO staff believe will be useful, instead of starting with technology projects conceptualized separately by outside researchers. Although other valuable frameworks have been used to evaluate technology products in healthcare settings, such as Normalization Process Theory (NPT) [19], the Nonadoption, Abandonment, Scale-up, Spread, and Sustainability (NASSS) framework [20], and the Consolidated Framework For Implementation Research (CFIR) [21], these theories and frameworks are focused more on issues related to implementation (e.g., why a new, existing technology was or was not widely adopted) than on design considerations for projects that have yet to be developed. Therefore, we have decided to conduct a needs assessment first and look forward to studying the implementation of technologies that may arise from our current work. At that time, we can employ important frameworks such as NPT, NASS, or a CFIR hybrid design [21] that can integrate an implementation science approach within studies of intervention effectiveness.

In keeping with the development steps and foci described above, we set out to conduct a needs assessment to examine how digital communication technology can potentially address some of the issues faced by CBO staff serving different PWUD populations. For this first step, we surveyed CBO staff, rather than service users, with the idea that subsequent phases of research would enable us to collect formative data from service users and would also enable us to work with service users to evaluate prototypes and/or multiple iterations of new designs [16, 22].

Details of our methodology and findings are presented in the following pages.

## Methods

Our research team conducted interviews with a total of 16 individuals, or stakeholders, who have experience working directly with vulnerable populations in both clinical and community settings. A combination of purposive and convenience sampling was used for recruitment, starting with a list of potential interviewees that was developed by the research team, in dialogue with two key informants or stakeholders, who are highly active in the national harm reduction community, work with harm reduction agencies, participate in harm reduction list serves, and attend national conferences.

### Sample

This final list contained names of 25 potential interviewees. The study team met to discuss these possible participants and were able to interview 16 stakeholders for the present analysis. The team was unable to connect with nine of the potential interviewees selected. Those interviewed were 7 female and 9 males, with age ranges from the early 20s through the mid-60s. Their work involved harm reduction agencies in the Southwest, Northeast, Midwest, including the Ohio Valley and Appalachia, and Southeast. All respondents have extensive backgrounds working in the field of harm reduction, providing low-threshold service to vulnerable populations including people who use drugs, sex workers, and homeless people. Stakeholders were located across the USA, in both urban and rural areas, and worked in harm reduction in service delivery, education, outreach, or programmatic areas. Stakeholders selected all had work experience with harm reduction agencies, in the past or at the time of the interview. Please see Table 1 for additional participant demographics.

**Table 1:** Stakeholder demographics

Gender	Age	Region	Urban/rural
Female	50s	Ohio Valley	Urban
Male	40s	Northeast	Urban
Male	40s	Northeast	Urban
Female	40s	Appalachia	Rural
Female	30s	East	Urban
Female	20s	Southwest	Rural
Male	20s	Southwest	Urban
Female	30s	Northwest	Urban
Male	60s	Midwest	Urban
Male	60s	Southeast	Urban
Female	30s	East	Urban
Female	50s	North	Urban/rural
Male	30s	Northeast	Urban
Male	40s	Midwest	Urban
Male	ND	South	Urban
Male	ND	Midwest	Urban

### Domains of inquiry

This stakeholder analysis aims to improve understandings of the unique programmatic and policy challenges and specific applications of technology in harm reduction settings. Stakeholder analyses have emerged as critical tools in policymaking, across political arenas and academic disciplines, and have become a particularly rich method within health policy research [23–28]. After agreeing to participate in the present study, stakeholders were interviewed either in person or over the telephone by two of the team’s experienced qualitative researchers. Domains of inquiry and related questions in the qualitative interview guide included the following:

- For what populations are technology-based interventions most useful?
- What is the optimal social and physical environment for technology-based interventions?
- For what purposes do you think technology-based interventions are best suited?

This focus on populations, places, and purposes allowed key stakeholders to speak to, and researchers to generate broad findings about, the ways in which technology might be deployed to benefit agencies and harm reduction programming and outreach. Based on their previous and current experiences, stakeholders were also asked to speak to the challenges, risks, costs, platforms, implementation, and barriers and facilitators to the effectiveness of technology-based interventions.

### Analysis

Interviews lasted between 20 min and one hour, and each respondent received a $40 honorarium in the mail for their participation. All interviews were digitally recorded, professionally transcribed, and checked for accuracy by research team members. The analysis involved an inductive and guided thematic approach. After an initial review of the transcripts, open coding was conducted to generate a preliminary code list. This list of emergent themes was then augmented with codes and themes based on the qualitative interview guide, until consensus on the final codebook was reached. Once the codebook was finalized, two researchers used the online Dedoose platform to code interviews. The research team met regularly throughout the data collection, analysis, reporting period of the project, discussing discuss preliminary results, and resolving any coding discrepancies.

## Results

Overall, stakeholders’ experiences varied widely regarding the type of and degree to which they used technology to develop and/or maintain relationships with clients, collect, organize, and analyze client demographic data, provide social services, and secure organizational funding.

### Potential impacts of use of technology

One of the most commonly desired impacts of technology on service provision was the ability to offer interactive educational and/or training materials to multiple participants at once, or to individuals who were only reachable via street outreach. One director at a grassroots organization operating a syringe exchange program in a densely populated urban area in NE USA noted the potential for materials designed to train individuals to properly recognize and reverse opioid overdose events can be augmented via technology assisted delivery:

The video should be like, hey, how does- how do you recognize a fatal overdose and is it current? How do you safely intervene? How do you administer Naloxone and then how do you call for help? I think those are kind of like the critical things, right? So like, “How to Identify”, “How to Safely Administer Naloxone”. “How to Perform CPR if Needed”, “How to Call for Help” … and I think like those are the key things that I think will matter. Oh, and then if the person might then there at the spot can be given Naloxone.

Similarly, an organizer with an all-volunteer harm reduction outreach service operating in the Southern United States suggested that mobile-based technology such as computer tablets preloaded with educational videos and other materials would be particularly useful for organizations who lack a physical office location and instead rely solely on street outreach:

Yeah, the first thing that I would put on there would be a guide to how to bleach clean your needles. And then, the next thing that I would put on there would be, like, more, um, safe injection information … so people understand how easy it is for them to get HIV.

One physician in a high-volume urban hospital further noted that the use of technology in providing educational materials aimed at encouraging behavioral changes might also encourage users to feel more comfortable learning about and discussing more sensitive subject matters:

It's like this intimacy thing … talking about sexual behavior and asking people to really think about making changes actually could be enhanced through technology because especially with millennials and younger people because of the comfort of having those conversations in the digital space. One of the barriers around sexual counseling has been the healthcare professionals’ discomfort. So I guess technology it would … potentially eliminate that.

For others, expanding their use of technology might simply entail increasing their use of social media in order to reach more vulnerable clients. A long-time director of a runaway and homeless youth shelter in a rural area of the Northeast noted that the use of social media enabled his agency to simultaneously connect with multiple unstably housed youth:

And so you know, we could be communicating with several different youths at once. I think that's the one thing with the phone is you're, you're on the phone with one person. I think sometimes the social media, you know, you can have a couple people going on and lay a little bit of groundwork and communication for when people show up to drop in, [anticipating] what they may need.

Despite often having only limited experiences utilizing technology-based materials, stakeholders noted that there are a number of ways technology might improve service provision for a variety of harm reduction, social service, and clinical settings. Specifically, technological resources might enable providers to reach multiple participants at once, as well as those with unstable housing, in a way that is allied with the harm reduction principals of meeting people where they are, on their own terms.

### Barriers to the use of technology in community-based or clinical service settings: data security and confidentiality

Although the stakeholders who were interviewed worked in a variety of harm reduction settings, from small, independent street outreach teams to larger, more formalized drop-in centers or clinical settings that use a harm reduction framework, they nonetheless shared similar concerns regarding the use of technology to engage with clients. Most stakeholders noted that historically, barriers to the use of technology in harm reduction settings have included intermittent Internet access, clients’ lack of technological proficiency, and the difficulties associated with providing staff with in-depth training. For street outreach and rural settings in particular, the lack of Internet access in remote areas proved to be one of the primary barriers to the use of technology, which stakeholders noted could be overcome by including downloadable apps or videos on cell phones or computer tablets.

For most stakeholders, however, the primary concerns related to the use of mobile technology in data collection and analysis centered on data security and client confidentiality. An organizer for an all-volunteer harm reduction organization in an urban area in the Southern United States noted that staff and clients alike often share these concerns:

Some people I've talked to, very validly, and it makes a lot of sense … are worried that that data can fall into the wrong hands and that's very sensitive information, obviously, um, because it does involve illegal activity, right?

Even with securely encrypted data, however, many stakeholders expressed concerns that clients might have reservations about using various technologies, particularly within the context of substance use and harm reduction. For instance, the long-time director of a semi-urban harm reduction-based outreach program noted the following:

The big issue when you're talking to people who are using illegal substances is the issue of trust. And that can go both ways. In some ways, um, you know, people base their sense of trust often on their gauge of the interaction with a person. Do you seem like somebody I can trust? And … you know, if you have a good feeling about somebody you're talking to and you feel like you trust them, then you might be more willing to give them accurate information or more in-depth information, um, or ask more questions and that sort of thing. If you're just presented with, um, computer tablets, then you have no idea who, who's on the other, who or what is on the other end of that and so you might be more reluctant to provide accurate or in-depth information or ask a lot of questions."

Relatedly, stakeholders frequently expressed concerns that those providing technology-based educational, training, or other materials face the additional challenge of being relatable, compelling, and trustworthy. For instance, a director working primarily with homeless individuals in a harm reduction setting noted the following:

So they can … access those types of things if that's something they're interested in watching. Um, I think it's just, just making them think its pertinent to their health, um also just recognizing the extreme, extreme kind of… what's the word I am looking for, just like problematic relationship between um, these folks, and perceived health care information just because of like a very stigmatized relationship that they have there. A lot of the time we really have to build quite a bit of rapport before anybody will talk to us about anything, especially if it's related to um, something like [HIV or HCV] testing. Nonetheless, that would be a lot easier because there's immediate benefit that like "I don't want my friend to die, and I've seen this happen so I want this."

Overall, stakeholders agree that despite acknowledging the potential advantages that technology-based materials might offer, developers and providers must address what they regard to be the primary obstacles to computer-based interventions, including concerns related to data security, lack of consistent Internet access, and unfamiliarity with certain aspects of emerging technologies.

### Adapting technology to client base

As noted above, most stakeholders repeatedly emphasized the importance of adapting technologies to meet the specific needs of the populations, places, and purposes with which they work in ways that were directly relevant to that group of individuals. For many, this meant adapting technology-based interventions not only with respect to race and gender, but with respect to regional and behavioral differences as well. One outreach worker at a mobile harm reduction agency in the Southern United States (introduced above) echoed the suggestions of many other stakeholders in suggesting that videos or other materials should be available in several population-specific iterations:

I would want the people narrating and demonstrating the videos to be someone who is a relatable figure to the person I'm talking to. Right? Like, I don't want a doctor explaining to somebody … I don't want anybody wearing, like, a suit. I don't want anybody with, like perfect English grammar. Like… And I also wouldn't want all of the representatives of the videos to be white.

Similarly, and particularly with respect to opioid overdose death prevention training, several stakeholders suggested developing technology-based materials specifically designed for individuals not only who are and who are not actively involved in substance use, but who may have community members or loved ones involved in substance use as well:

I definitely see that there could be a huge benefit for having, these, these [different people] talking about Narcan, like, having these sort of tailored, um, videos that people are able to watch, in here with people that are in communities with them as well. I mean there's a type of video that would make a lot of sense for people who are using drugs and there's a type of video that would make a lot of sense for, like, concerned mom who, like, knows four or five other concerned moms to sit down and watch together.

Other stakeholders suggested that materials not only feature individuals with whom the intended audience relates or identifies, but that make direct reference to specific agencies or organizations as well. For instance, one staff member at an SSP serving runaway and homeless youth in a large, metropolitan area suggested that videos and other materials include information designed to introduce the organizational staff to potential clients:

If [our agency] had its own specific, like, overdose prevention video and you could see, like, what staff looks like and how we approach the work. If it was, like, something that was, like, tailor-made by the organization, that I think you could get a feel for the organization. Um, if it was like for fundraising purposes, like we could share the video on social media and people would like get to know a little bit about street work and who we are. Yeah. I think it would be different if it was like department of health made this video and everyone has to use it kind of thing.

Finally, several stakeholders suggested that mobile and other technologies should also be adapted to meet the needs of the staff:

And like, after street outreach, people go home and then have to bring all of that paperwork back to the drop-in, which is sometimes tricky, right? Um, and I think about like when we do outreach like in the rain or like at an event, how that makes it even more annoying to like have to bring back nice pretty stat sheets to the drop-in. Um, so I think it could be helpful to have like some sort of technology, something helping us do outreach. Um, but I could also see how like people would leave tablets at home or, you know, so I think there's always like, challenges in that.

Overall, stakeholders were clear that in order to be effective technology-based interventions must be adapted to meet the specific needs of both clients and staff rather than vice versa.

### Technology versus face-to-face interactions

In addition to being specifically adapted to meet the needs of clients and staff, most stakeholders also suggested that technology-based interventions should be developed with the aim of facilitating and augmenting face-to-face provider–client interactions rather than replacing the latter altogether. One program director at an urban, mid-Atlantic overdose prevention program expressed this often repeated concern:

We've used training videos, um, at the needle exchange site when we're seeing a lot of people, training a lot of people on naloxone … But we didn't just leave the people alone in the room with a video. We had someone there who could answer any questions, review, uh, you know, and, um, address any concerns, that sort of thing. I do think the human contact is important.

For other stakeholders who shared these concerns, however, the ability to develop and maintain face-to-face relationships with a client base they consider to be increasingly technologically oriented often required utilizing social media, mobile devices, and other technology-based outreach to engage initially. One director at a large runaway and homeless youth organization serving urban youth in the Northeast noted that many clients are frequently unable or unwilling to engage with service providers until contact has been initiated via phone or computer:

I get the need for human action- interaction but the reality is that young people are only getting that once they get to the program. And what I'm worried about, you know, and what we've been worried, like… what happens to all the young people that never make it to the programs? What happens to all the young people that call a service provider four times and no one answers the phone and they don't try again? … This is the gener- the generation under me. Like, they're all technology based. But that's how they function in the world. They don't even know how to call people.

Similarly, the director of a homeless youth drop-in center in a mid-sized city on the East Coast suggested that by adopting a human-centered approach to technology, many harm reduction or other social services agencies might benefit from using various technologies to enable staff to sustain contacts with young clients in particular:

If you were going to have something at some point where there's a real connection, like a live chatting or communicating in some form, you have to have staff time for that. You have to see, and I think also it's adjusting our mindset that social media just isn't in your development department. Right? … It’s really a way to stay connected to young people so, we have to actually think about it in a kind of caseworker, counselor, outreach worker kind of way … you need to create the engagement and then once you have an appropriate engagement, then I think technology would become a lot more useful.

Stakeholders were particularly interested in exploring the possibility of using technology to reach existing or potential clients in rural areas where public transportation is often inaccessible. Several individuals interviewed noted that physically accessing an SSP or a drop-in center is often financially or otherwise prohibitive for many of those in need of services, and suggested that technology-based interventions may offer a viable alternative to face-to-face interactions when necessary. Citing limited resources such as transportation and staff, the director of a harm reduction-based drop-in center and outreach program serving multiple locations surrounding a metropolitan area in Appalachia noted the following:

We provide training on how to use naloxone. We go out in the community settings and, uh, for a long time, we were the only people who were doing those trainings and we really had one staff person who could do that, who could travel to various places and do those trainings. So, as videos were developed to provide that training, uh, we could, you know, we could tell people, you know, places where we you'd have to, you know, we would have to drive three hours to do the training. We could say, "We can't come there, but here is a, now here's a training video or … some other kind of training module, interactive kind of training module, um, that would, uh, replace having to have a second staff person to drive long distances to do those trainings.

Likewise, an outreach staff member at an all-volunteer harm reduction program located in the Southern United States and serving several large rural areas suggested that being able to remotely provide clients with videos, text messages, and other technology-based materials prior to contact might aid in providing services in remote locations:

It would be pretty helpful for me to be able to, before I make it out to a site, bring somebody information on, like, "hey, we could do this for you,” like, very quickly and easily … so I wouldn't have to like, explain over text again and again to somebody just because, um, in so many of these areas, … the level of education of like understanding spread of hepatitis B and HIV, um, and, like safe injection practices is very, very low. Like, very bad understanding. Like, not good education. Like there's not even good sex education out here, much less safer drug use education.

Overall, while stakeholders agreed that technology-based interventions should not be used as a substitute for face-to-face client–provider interactions, there are nonetheless myriad circumstances wherein other modes of engagement are simply not feasible. The director of a harm reduction-based outreach program and drop-in center (introduced above), for instance, noted the following:

On the other hand, there are circumstances where, where people are in a remote location. There's no one there that is available to do a training and so they might just watch a training online and that will be, that's better than nothing. So I guess that's a balance. I mean, I think ideally, you wanna be able to have access to human contact as well but in circumstances where it's not available, um, it could supplant it, I suppose.

While stakeholders concurred that face-to-face provider–client interactions are and should remain the gold standard, many nonetheless acknowledged that in locations wherein there is little or no possibility of face-to-face support services, technology might still work to encourage or maintain an element of connectivity, with the possibility of leading to face to face in the future.

### Providing up-to-date information regarding local resources

One of the most important service provision gaps that stakeholders cited was the lack of access many clients and staff have to up-to-date information regarding local resources available to those who need them. During the early weeks of the COVID-19 crisis, this need for up-to-date information was particularly salient as hospitals in some cities were bursting at the seams and sending patients away, and food and other supplies became more and more scarce. Many of those interviewed expressed concern that even upon establishing relationships, clients and staff are frequently unaware of the almost constantly shifting landscape of local and regional social services and other, less formal resources. The director of a large, urban runaway and homeless youth organization recalled that many of her agency’s clients had already expressed a keen interest in technology-based materials providing real-time information regarding local resources and opportunities:

One of the things that the young people want to do that I think is an amazing idea is to create an app that goes broader than just, um, connecting young people with formal resources in real time and really gets to the heart of what it means to like, support each other as a community, especially from a harm reduction [stand]point … something similar to Yelp, actually, um, where young people can put in their address and then anything that there's near them will pop up through the GPS feature … the things that will make it really great, and especially like, young people friendly, is the young people would have the capacity to leave reviews for the programs. Um, that there would be some kind of a chat or like a blog feature so that young people could share informal resources, so things like "Hey, if you go to the youth stop on the [train] between 10 and midnight, like the guy's cool, you jump in the turnstile." But like nothing the city could ever have on something that they put but those things are important, right? Like "Hey, this [coffee shop], like he'll let you sleep there if you buy a cup of coffee," you know, like free things like that, um, so I think that's what they're looking to do, um, that obviously would have to be done under a… a non-government entity.

Others noted that the ability to access information as simple as which homeless shelters have open beds, which drop-in centers are open during which hours, where naloxone is free and available, or even which substances might be cross-contaminated might be invaluable to those seeking immediate assistance. For instance, the director of a youth-based syringe exchange program and drop-in center in a large metropolitan area stated the following:

I think it would be cool to have like a resource app or page or whatever portal, I don't know, it could take so many forms, like where young people could like look-up whatever resource they wanted.[…] Like, something like that would be cool if the person was like, "Oh I need to find a shelter for a 25-year-old whatever person," you know, like where could you send me? Or if you could look-up like 24-h drop-in centers or, um, like general substance use harm reduction or like different resources in one area. Yeah, yeah. You can filter by zip code or whatever. I think that would be cool. And then to have some sort of like messaging or like email function to where you could, like if you access [an organization], you could email your case manager or you can, if you access [a local clinic] you can access your test results or, you know, something like that. That would be cool.

Indeed, the need and interest for more accurate and up-to-date resource hubs for both clients and staff was a common theme throughout these interviews.

## Discussion

The present study sought to elicit perspectives on technology from stakeholders who have extensive experience working with vulnerable populations in harm reduction settings. As detailed above, responses to our interview questions fell into a handful of general areas. In terms of benefits, respondents noted the potential to deliver consistently high-quality education content to multiple clients (e.g., training individuals to recognize and respond to an overdose event). This is a highly straightforward use of technology with clear benefits that have been written about previously—for example, some SSPs rely on volunteer staff, and technology-based tools can enable volunteers to deliver high-fidelity intervention content, even if a volunteer might lack extensive training or experience [10]. A respondent also noted that, in some cases, people may prefer addressing sensitive issues (e.g., discussions of sexual risk behaviors) via computer, instead of face to face, because the computer may lessen, or eliminate, “discomfort” on the part of clients and/or staff. This has emerged frequently in research of technology in healthcare settings (e.g., Mackenzie et al. [29] and Marsch et al. [30]): Participants often do not fear that a computer will judge them if they report risk, but fear that a person might.

Stakeholders also discussed the value of being able to keep in touch with clients remotely. These remote communication projects may prove especially valuable for organizations that serve isolated clients in rural areas, as well as CBOs in urban locales seeking to serve clients during shelter-in-place situations. Some, such as an online “bad date list” [31, 32] or messages designed to alert clients to especially deadly batches of drugs being sold in an area can be readily delivered using inexpensive technologies that substance using clients likely already have access to, such as a pre-formatted Web page, a group text, or free downloadable mobile phone applications such as WhatsApp. Recent research indicates PWID frequently have mobile phones and respond quickly to text-based prompts [33]. Text messages can be a particularly good way to reach people because they do not require special equipment (beyond a mobile phone) or advanced training to use. A significant issue with text-based communication is that texts are by definition not secure and could potentially be read by people other than the intended recipient, including law enforcement. WhatsApp offers encrypted communications that are more secure, but still may not be entirely private—the application is owned by Facebook. (Additional data privacy issues are discussed below.)

Other uses of technology that emerged as themes in our interviews would require a greater investment of resources. Stakeholders discussed potential benefits of customizing or tailoring content so it becomes more “relatable” to clients in specific settings, or geographic areas, or to members of specific population groups (one respondent quoted above discussed not wanting someone to appear onscreen wearing a suit or using speaking in “perfect English grammar”). These types of projects would require more time and funding to develop, but may also prove highly worthwhile. As Bandura describes in Social Cognitive Theory, the perceived relevance of intervention messaging is chiefly important—before intervention recipients will take action to change any behavior, they must first decide that the behavior, as well as the intervention content, are worth attending to [34]. Of course, “relatable” and “relevant” can mean different things to different people. Bandura [35] as well as Fisher and Fisher [36] posit that intervention materials must be made relevant to specific population groups and the problems they face. Interestingly, findings from the current stakeholder analysis align with prior research suggesting it may prove just as important for technology-based interventions to be attentive to behavioral distinctions (e.g., different types of substance use) as it is to match the ethnic, racial, or gender makeup of participants to people onscreen [37]. In other words, making “relatable” content may be less about what the people on a screen look or sound like, than about how closely the behaviors they depict or discuss match those of the audience.

Clear barriers that emerged from our interviews include data security, and the need to maintain the trust of clients. These are well-founded concerns, especially when using existing technologies such as Facebook, SurveyMonkey, and products such as Google Forms or Gmail. Personal data can be bought and sold, so anytime an organization is using technology to collect, store, or transmit data, privacy becomes an important consideration. One solution is to create applications that never collect or store any identifiable personal data. Another is to use some type of confidential identifier (i.e., a number or a nickname) and to store any linkage data (the “key” that can link a confidential ID to a client’s actual identity) separately from the computer application, in an encrypted format or possibly even in non-digital form (e.g., printed paper in a notebook), in order to lessen the chances client privacy may be compromised.

Law enforcement or other authorities can potentially identify clients who access an application by their IP address (the numeric identifier of a specific Internet connection, e.g., the address of the Internet connection in a person’s home) or by a unique device number such as an IMEI used to identify mobile phones. One way people have worked to address these issues is to create applications that never capture identifying data such as names or addresses, and then to load these applications onto tablets that clients can use to access interventions, which are connected to a mobile Internet hotspot. This way, no data are traceable to any individual who participates in a project.

Incorporating this level of privacy protection into an application creates an additional level of work, but enables far greater security. If you are using prepackaged software, especially something you do not need to pay for, it can be very hard to make sure your data are being used in ways that you find acceptable. Just as if you make a cake from a mix, to go back to the analogy from Introduction, it might contain ingredients that you would otherwise not find acceptable to eat or to serve to people you care about. These issues of privacy have become particularly salient in the era of COVID-19, as people are using technology to reach clients they cannot meet with in person for safety reasons. Video conferencing can be very convenient, but it may not be secure. How do you know who is monitoring your conversations, especially if you may be discussing sensitive information (i.e., drug use)?

An additional concern raised by a number of respondents is the need for human interaction, especially in harm reduction settings, and that technology not be used to replace face-to-face contact. Well-implemented technology projects can enable staff to work more effectively with larger numbers of people (e.g., offering HIV/HCV testing in ways more clients will accept, or using tablet computers to conduct private automated behavioral screenings with multiple clients at once), and need not be designed to take the place of CBO staff. Developing technology that emphasizes human connections may take on greater importance due to social distancing measures that will likely remain with us for the foreseeable future. If people cannot meet face to face, the ability to remain in contact via technology may prove even more important. If done well, technology may enable SSP and other CBO staff to build upon existing interpersonal relationships and help combat feelings of loneliness and isolation, especially among people with compromised immune systems or other health conditions who may not be able to travel to drop-in centers or outreach sites.

A further barrier to technology use is a lack of Internet, or a lack of broadband Internet with adequate capacity, that would enable the use of technology-based projects in rural or potentially underserved urban areas. This can potentially be addressed by creating media content that is preloaded, or *cached*, on a device and then played back without accessing the Internet. In situations where a device is used to collect data in an area without Internet connectivity, these data can be temporarily cached on a device and then uploaded to an online server when an Internet connection becomes available. (Applications can be programmed to check for an available connection to a server, or the upload can be initiated manually.) Again, technology can be designed and developed to fit specific situations and circumstances.

Lastly, the greatest barriers to technology use may be cost and access—many CBOs are staffed by volunteers or operate on budgets that leave little or no money for technology development. Moreover, if service recipients are focused on day-to-day survival, they likely won’t have funds to acquire fancy new digital devices, nor will they have time to learn how to use them. These barriers are real, but not insurmountable. We suggest there may be ways to develop affordable technology that can be readily integrated into CBO workflows. Our goal in documenting the needs of CBO staff and leadership is to facilitate conversations that lead to worthwhile, low-cost technology-based solutions. Focusing on principles of effective design, including processes of ongoing evaluation and revision in response to end-user feedback, can potentially yield products that are easy for staff and clients to use without extensive training [10, 22], and also produce measurably improved outcomes.

## Limitations

The greatest limitation of the current survey is that was conducted solely in the USA and therefore did not collect viewpoints of providers doing valuable work in other parts of the world. Another limitation is that, as mentioned in the introduction, we chose to first assess the needs of service providers without interviewing clients. We hope to address both in future research and consider the current paper an important first step, not a last word.

## Conclusion

As these interviews illustrate, technology is by no means a cure-all, and often entails clear issues (particularly in relation to cost, data safety, and privacy) that must be addressed before it can be used safely. Nonetheless, technology also offers opportunities to serve people who otherwise might not be reached, and to customize content for specific populations. In this era of COVID-19 social distancing, the ability to maintain contact remotely and to deliver reliably high-quality information to isolated clients offers important possibilities for SSPs and other CBOs. The human-centered approach to technology development that we describe in the introduction aligns strongly with a client-centered approach to harm reduction. Both emphasize making the most of available resources to serve people without judgment. Given the current healthcare situation, they may provide more valuable opportunities now than ever before.
